# Treatment of Trigeminal Neuralgia with Anti-viral Therapy for Zoster: A Case Report

**DOI:** 10.7759/cureus.2144

**Published:** 2018-02-02

**Authors:** Andrew R Spector

**Affiliations:** 1 Department of Neurology, Duke University Medical Center, Durham, NC

**Keywords:** varicella zoster virus, vaccines, hhv-3, zoster sine herpete, trigeminal neuralgia

## Abstract

Varicella zoster virus can infect the trigeminal ganglion, but viral infection is not frequently considered as a cause of trigeminal neuralgia. This is a case of a woman whose trigeminal neuralgia remitted after being treated with valacyclovir for a thoracic zoster rash and later resolved entirely following administration of the zoster vaccine. Successful treatment of trigeminal neuralgia with anti-viral therapies has not previously been reported. Zoster vaccination is currently recommended for adults over age 60, so patients with trigeminal neuralgia, especially those over the age of 60, should be encouraged to receive the varicella zoster vaccine.

## Introduction

Varicella zoster virus (VZV) was first isolated from trigeminal ganglia in 1983 [1, 2]. It is well established that zoster outbreaks along the trigeminal nerve cause acute and chronic pain. In some cases, pain has occurred without the rash and is still attributed to VZV (zoster sine herpete, ZSH) [3]. Differentiating ZSH from idiopathic trigeminal neuralgia is clinically challenging and requires serological testing to identify a sudden increase in VZV antibodies, polymerase chain reaction (PCR) of the cerebrospinal fluid to identify circulating virus, or post-mortem examination of the trigeminal ganglion. Clinical history can be helpful in that ZSH is thought to be more continuous, similar to post-herpetic neuralgia, rather than presenting with intermittent attacks as is often seen in trigeminal neuralgia. The early studies identifying VZV in trigeminal ganglia were conducted on subjects without known trigeminal distribution pain [1, 2]. No study directly identifying VZV in the trigeminal ganglia of living patients with typical trigeminal neuralgia and no rash was identified by literature review.

## Case presentation

An 81-year-old healthy woman presented to a general neurology clinic in 2014 for a second opinion regarding pain on the left side of her face. She described the pain as electric shocks in her cheek from below her orbit to her chin. The pain was only present on the left and could be triggered by eating, brushing teeth, and other mouth movements. The pain was severe and lasted 20-30 seconds at a time. The pain came in cycles lasting about three months each. Her first cycle was in 2006, and there was no preceding injury or associated rash. When she presented in 2014, the cycle was not resolving spontaneously and the pain was more intense than previous cycles. She had no improvement with acetaminophen with codeine or tramadol. Her neurological examination was unremarkable with the exception of mildly decreased pinprick sensation in the V2 distribution on the affected side. Her previous neurologist prescribed carbamazepine, but she never tried it due to concerns over listed side effects. She was evaluated with magnetic resonance imaging (MRI) of her brain and magnetic resonance angiography (MRA) of the head. No structural or vascular etiology of her symptoms was identified.

She was given a diagnosis of idiopathic trigeminal neuralgia and started on gabapentin as a more palatable alternative to carbamazepine. Gabapentin helped her pain but led to weight gain, flattened affect, impaired memory, and poor coordination. She weaned herself off gabapentin prior to her follow-up visit. At that time, her pain had resolved, so she opted not to continue any prophylactic medications. She continued to have cycles of pain every one to two months for which she was given a prescription for low-dose gabapentin to be used as needed.

In May 2016, she developed on outbreak of zoster on her torso. She was treated for this with valacyclovir with good results, and she did not develop post-herpetic neuralgia. She observed that her facial pain diminished during antiviral treatment and returned upon completion. At her husband’s urging, she was vaccinated for the first time with Zoster Vaccine Live (Zostavax, Merck & Co. Inc., NJ, USA) in July 2016. She noticed that over the subsequent week, her left cheek pain gradually resolved, and at the present time, it has not recurred. This is now the longest period she has gone without trigeminal pain since 2006.

## Discussion

Approximately 11% of trigeminal neuralgia remains idiopathic following clinical and radiological evaluation [4]. This is the first report of idiopathic trigeminal neuralgia improving with antiviral therapy and resolving with vaccination for zoster. The presumed mechanism for this is suppression of an underlying varicella zoster viral (VZV) infection of the trigeminal nerve. As there was no antecedent rash, this could be a case of zoster sine herpete (ZSH). However, her clinical course with pain starting 10 years before her zoster rash and spontaneous remissions lasting months at a time is more similar to trigeminal neuralgia. Regardless, with no good way to make a prospective diagnosis of ZSH, a viral etiology should remain on the differential for patients presenting with idiopathic trigeminal neuralgia.

It remains to be seen how long this patient’s clinical benefits will last or if repeat vaccination will provide additional benefit in the future. It is also not known if valacyclovir therapy alone could have produced a lasting remission had she been treated for a longer period or if it could be used to treat pain as needed on a recurring basis as in herpes labialis outbreaks.

It is not suspected that all patients with trigeminal neuralgia would respond to zoster vaccination as there are multiple known non-viral etiologies for the condition (e.g. vascular, demyelinating), but vaccination is an option, already recommended for adults over age 60 based on current guidelines and presumably safe for adults of younger ages also [5]. For Zostavax, caution should be exercised for those with abnormal immune systems as it is a live vaccine, but the currently recommend shingles vaccine, Shingrix, (GlaxoSmithKline plc, Brentford, United Kingdom) is recombinant and available for immunocompromised patients [6].

## Conclusions

Given the severity of pain experienced by patients with trigeminal neuralgia and the potential toxicities, complications, and inefficacies of known therapies, zoster vaccination would be a welcome addition to the armamentarium of treatment options. Treatment of idiopathic trigeminal neuralgia with viral suppressing therapies should be considered.
